# Supplementary material for: Investigating approach/avoidance tendencies in male AUD patients through a gait initiation task: An exploratory posturography study
Source: PLoS One. 2025 Aug 13;20(8):e0327765. doi: 10.1371/journal.pone.0327765 (PMC12349135; doi:10.1371/journal.pone.0327765)
Supplement: Attachment — Submitted filename: ResponsesToReviewers_auresp_2.docx [file pone.0327765.s003.docx]

**We thank the Editor and both reviewers for their thorough job which helped us a lot to increase the quality of our paper. We hope you will be satisfied by our modifications, and we remain at your full disposal for any further requirement. Modifications can be checked at pages reported below in the MS with tracked changes (PlosOne_Revision2_Edited.doc).**

**Reviewer #1**: The authors have addressed my comments and provided a corrected revision. I have no further objections. Thank you for sharing your interesting findings!

*Thank you very much.*

**Reviewer #2**: The authors have thoroughly revised the manuscript, which is now much clearer, more methodologically rigorous, and easier to follow.
I have only one final comment for the authors, which concerns one of the early points in the Discussion section. Specifically, the authors state that "participants of all groups were faster initiating a forward movement... suggesting an alcohol avoidance bias." However, the results show a main effect of stimulus type, but not an interaction with movement direction. Therefore, I do not believe it is appropriate to interpret this as evidence of an avoidance bias. Rather, the data seem to indicate a general slowing in response to alcohol-related stimuli, regardless of movement direction.
Apart from this, in my view the paper is now ready for publication.

*We thank Reviewer 2 for this comment. This was updated at page 17.*
